# Milnacipran continuation during pregnancy in a patient with severe post-traumatic stress disorder and psychotic depression: a case report with 3-year pediatric follow-up

**DOI:** 10.3389/fpsyt.2026.1830520

**Published:** 2026-04-27

**Authors:** Maximilian Preiss, Lucie Bartova

**Affiliations:** 1 Department of Psychiatry and Psychotherapy, Division of General Psychiatry, Medical University of Vienna, Vienna, Austria; 2 Comprehensive Center for Clinical Neurosciences and Mental Health, Medical University of Vienna, Vienna, Austria

**Keywords:** depression, hyperemesis gravidarum, maternal safety, milnacipran, pregnancy, suicidality

## Abstract

Evidence on milnacipran exposure during pregnancy is sparse, complicating treatment decisions when maternal stability depends on this antidepressant. We report a 31-year-old primigravida with severe post-traumatic stress disorder and a severe major depressive episode with psychotic features in the context of a chronic, difficult-to-treat psychiatric course marked by self-harm, dissociation, and recurrent suicidal behavior. Milnacipran 150 mg/day led to marked clinical improvement before pregnancy. After pregnancy confirmation, treatment was changed to sertraline plus quetiapine because of limited pregnancy safety data, but depressive symptoms and suicidal behavior reemerged within 2 weeks. Milnacipran was therefore re-established after risk-benefit evaluation and shared decision-making, with renewed improvement. Moderate hyperemesis gravidarum during the second trimester led to discontinuation of milnacipran by gestational week 22. Delivery occurred at gestational week 38 via primary cesarean section. No congenital anomalies or neonatal adaptation problems were observed. At 3-year follow-up, pediatric developmental screenings remained within expected limits. This case illustrates the challenge of balancing relapse risk, prior treatment response, and treatment feasibility during pregnancy when evidence for the effective medication is limited.

## Introduction

Pharmacological treatment of depressive and anxiety-spectrum diseases during pregnancy remains a frequent and clinically consequential challenge. Antidepressant discontinuation in pregnancy is common and may carry a clinically meaningful relapse risk, particularly in women with severe, recurrent, or difficult-to-treat illness (1–3). Clinical decisions about switching or discontinuation are further complicated by pregnancy-related symptoms that may affect medication tolerability and adherence, while reproductive safety data remain robust for only a limited number of antidepressants (4–7). Recent population-based work furthermore highlights that perinatal morbidity and mortality are strongly shaped by behavioral health trajectories. This especially includes women where suicidality and self-harm may accompany depressive worsening (8, 9).

Milnacipran is a serotonin–norepinephrine reuptake inhibitor for which pregnancy safety data remain sparse. The largest identified source of pregnancy-specific data is the Savella Pregnancy Registry, a prospective US exposure registry that enrolled only six pregnant patients between 2009 and 2024; five pregnancies resulted in six live-born infants, one pregnancy outcome was lost to follow-up, and the final report concluded that available data were insufficient to determine an association between maternal milnacipran exposure and premature birth or birth defects (10). As with other less commonly used psychopharmacotherapeutics, this leaves clinicians and patients to balance maternal benefit against uncertain fetal and neonatal risks under conditions of incomplete evidence.

Here, we report a case of severe, chronic, difficult-to-treat psychiatric illness in pregnancy in which switching from milnacipran to a better-studied regimen was followed by relapse, and milnacipran was subsequently resumed after shared decision-making. This case highlights the clinical challenge of balancing relapse risk, prior treatment response, and treatment feasibility during pregnancy when evidence for the effective medication is limited.

## Case report

This case report is based on routine outpatient psychiatric documentation, contemporaneous clinician notes, patient-reported information, and available obstetric and pediatric follow-up records, including entries from the *Eltern-Kind-Pass* (Austria’s standardized parent-child health record). Diagnoses were established in routine clinical care according to ICD-10 criteria. Data on medication exposure, treatment changes, symptom course, pregnancy outcome, and child development were collected retrospectively from these records and a patient interview. Standardized symptom rating scales beyond the Clinical Global Impression Severity scale (CGI-S) were not routinely administered in this outpatient setting. Written informed consent for publication was obtained from the patient. For a timeline overview, see **Table 1**.

**Table 1:** Timeline of treatment progression and outcome.

Time point	Gestational period	Clinical status	Treatment/intervention	Outcome/observations
Oct 2020	Pre-pregnancy	Onset of severe PTSD and MDD with psychotic features; dissociation, self-harm, suicidal strangulation attempts	Psychotherapeutic, chronobiological, and pharmacological interventions	Persistent severe illness; CGI-S 6/7
Oct 2020–Apr 2022	Pre-pregnancy	Difficult-to-treat course with minimal improvement	Several antidepressive and adjunctive treatment regimen	No sustained clinical improvement
May 2022	Pre-pregnancy	Severe depressive, dissociative, and suicidal symptoms	Milnacipran initiated and titrated to 150 mg/day; levothyroxine 150 µg/day; eszopiclone 1 mg PRN	Clinical improvement; cessation of self-harm and suicidal behaviour; CGI-S 2/7
Aug 2022	Pregnancy confirmed; GW4	Clinically improved on milnacipran	Switch to sertraline 100mg/day + quetiapine 25mg/day and up to 100mg PRN	Pregnancy continued
Sept 2022	GW6	Reemergence of depressive symptoms and suicidal behaviour	Re-initiation of milnacipran 150 mg/day	Renewed improvement; CGI-S 2/7
July-Oct 2022	1st trimester	Mild nausea and gastritis	Doxylamine 10 mg/day + pyridoxine 10 mg/day; pantoprazole 40 mg/day	PUQE 4/15; Nausea controlled
Oct-Dec 2022	2nd trimester; GW14-22	Gradual increase to moderate hyperemesis gravidarum; emesis 3–4 times/day	Antiemetic treatment ineffective; management with general internist	PUQE 12/15; no severe complications
Dec 2022	GW22	Persistent hyperemesis and poor oral tolerability	Abrupt discontinuation of milnacipran, levothyroxine, and eszopiclone (oral intake resulted in emesis)	No withdrawal symptoms documented; BP and thyroid status stable
Dec 2022- Feb 2023	2nd/3rd trimester; GW22-28	Hyperemesis gradually improved	No further specific intervention reported	Pregnancy continued without major obstetric complications
Apr 2023	Delivery; GW 38	No clinically significant depressive symptoms or active suicidality documented at that time	Primary cesarean section (elective indication at patient request)	Male newborn; 3455g/52cm; Apgar 9/10/10; discharge after 3 days
Apr-Nov 2023	Postpartum/lactation	No postpartum depression or PTSD symptoms documented during this period	Breastfeeding for 7 months; milnacipran not resumed postpartum	Child developmental screenings remained within expected limits
Mar 2025	2-year follow-up	—	Routine pediatric follow-up	Child developmental screenings remained within expected limits
Mar 2026	3-year follow-up	—	Routine pediatric follow-up	Child developmental screenings remained within expected limits

BP, blood pressure; CGI-S, Clinical Global Impression Severity scale; GW, gestational week; MDD, major depressive disorder; PRN, as needed; PTSD, post-traumatic stress disorder; PUQE, Pregnancy-Unique Quantification of Emesis and Nausea score. Child developmental outcomes were based on routine pediatric follow-up, including documentation in Austria’s standardized parent-child health record. “No severe complications” indicates no hospital admission, intravenous fluids, ketonuria, or other major obstetric complications. “No clinically significant depressive symptoms or active suicidality documented” refers to routine clinical documentation at the respective time point.

A 31-year-old woman with no substance use (no smoking, alcohol, or illicit drugs) and normal weight at pregnancy onset was treated for a severe post-traumatic stress disorder (ICD-10 F43.1), complicated by a severe major depressive episode with psychotic features (ICD-10 F33.3). The illness began in October 2020 after a traumatic bereavement (death of her brother) and was characterized by a high burden of symptoms including intense fear of loss, severe social anxiety and depression, agoraphobia, dissociation, self-harm, and recurrent suicidal strangulation attempts. Functional impairment was substantial and clinician-rated severity was described as high (CGI-S 6/7).

Over the course of illness, multiple psychotherapeutic, chronobiological (bright light therapy), and pharmacological strategies had been attempted, consistent with a difficult-to-treat trajectory (11, 12). Prior medications included antidepressants such as sertraline, venlafaxine, vortioxetine, mirtazapine, trazodone, agomelatine, tianeptine, as well as adjunctive agents (including quetiapine, cariprazine, olanzapine, aripiprazole, chlorprothixene, pregabalin, and lorazepam). All medications were titrated to therapeutic dosages and mostly discontinued after at least 4 weeks because of minimal or no clinical improvement.

In May 2022, milnacipran was initiated and, because it was well tolerated, titrated to 150 mg/day resulting in marked improvement and cessation of self-harm and suicidal behavior, as well as depressive and dissociative symptoms (CGI-S 2/7). Mild adverse effects included brain fog and sedation, which the patient accepted in view of the substantial clinical improvement. Concomitant medication included levothyroxine 150µg/day for comorbid Hashimoto’s thyroiditis and eszopiclone 1mg as needed for insomnia.

In July 2022, the patient became pregnant unexpectedly while on treatment. After counseling about the limited evidence for milnacipran in pregnancy and a shared risk-benefit discussion, the medication was changed to a regimen of sertraline 100mg/day and quetiapine 25mg in the evening as well as up to 100mg as needed. However, after 2 weeks depressive symptoms and suicidal behavior reemerged (CGI-S 5/7). The patient, together with her treating psychiatrist, chose to reestablish milnacipran at 150mg/day, which was followed by renewed improvement within two weeks (CGI-S 2/7). At the beginning of pregnancy, the patient experienced mild nausea (PUQE score 4/15) and gastritis. Nausea was sufficiently treated with a combination of doxylamine 10mg/day and pyridoxine 10mg/day, the gastritis was treated with pantoprazole 40mg/day. During the second trimester, nausea worsened to moderate hyperemesis gravidarum, with emesis occurring 3–4 times daily (PUQE score 12/15) (13). The antiemetic by then was reported as largely ineffective. Management was provided in collaboration with a general internist. There were no hospital admissions, no intravenous fluids, and no ketonuria reported. Because of persistent hyperemesis and poor tolerability of oral intake of any medication, milnacipran, levothyroxine, and eszopiclone were abruptly discontinued at the patient’s request by gestational week (GW) 22 without withdrawal symptoms. Blood pressure remained within normal limits at routine check-ups, and thyroid status remained stable on serial blood tests. From GW22 to GW28, symptoms of hyperemesis gravidarum gradually improved and ultimately resolved without further intervention.

Delivery occurred at GW38 via primary cesarean section (elective indication at the patient’s request). The male newborn had a birthweight of 3455g, a length of 52cm, and Apgar scores of 9/10 at 1 min, 10/10 at 5 min, and 10/10 at 10 min. The only somatic issue was transient neonatal jaundice. No congenital anomalies or neonatal adaptation problems were observed. The newborn was not admitted to a neonatal intensive care unit, and both mother and child were discharged 3 days postpartum. Breastfeeding continued for 7 months; milnacipran was not resumed postpartum. This decision was made jointly with the treating psychiatrist, as the patient remained clinically stable and had experienced mild but unpleasant adverse effects. In this context, resuming milnacipran solely for prophylactic purposes during lactation was not considered justified given the limited evidence for its use. At 3 years of follow-up, routine pediatric developmental screenings remained within expected limits (latest check-up in March 2026). Postpartum and during lactation, the mother had regular psychiatric checkups and, despite the heightened risk of postpartum depression, remained clinically well and stable, with no symptoms of depression or PTSD.

## Discussion

This case adds to the limited clinical experience regarding milnacipran exposure during pregnancy. It is notable both for the clear temporal sequence in which switching from the only clearly effective antidepressant was followed by clinical deterioration, whereas re-establishing milnacipran was followed by renewed improvement, and for the availability of pediatric follow-up to 3 years. This temporal sequence underscores the relevance of prior treatment response and maternal relapse risk when making pharmacological decisions during pregnancy (1). The central clinical problem was the need to balance the maternal benefit of maintaining an antidepressant that had been uniquely effective in a difficult-to-treat course against uncertain fetal and neonatal risks in the setting of sparse pregnancy outcome data (2, 4). The available evidence base for milnacipran in pregnancy remains very limited. The Savella Pregnancy Registry, the largest identified pregnancy-specific data source, enrolled only six pregnant patients and yielded insufficient data to assess associations with prematurity or birth defects (10).

Several considerations limit inference. Milnacipran was discontinued by gestational week 22; therefore, this case provides only limited information about late-gestation exposure, when neonatal adaptation symptoms are most often discussed for serotonergic agents (5, 14). Likewise, no conclusions can be drawn regarding lactational exposure, as milnacipran was not resumed postpartum. Although no congenital anomalies or neonatal adaptation problems were observed and pediatric follow-up remained reassuring, child development was assessed through routine pediatric screening rather than standardized neuropsychological testing, which would have provided greater precision (7). In addition, concomitant early-pregnancy exposure to eszopiclone further limits any causal interpretation of the absence of adverse findings in relation to milnacipran alone. As with any single case report, generalizability is necessarily limited.

A further clinically relevant point is that hyperemesis gravidarum became a major determinant of treatment feasibility. In this case, antiemetic treatment did not adequately control symptoms, and tolerability of oral medication progressively worsened, ultimately leading to discontinuation of milnacipran in mid-pregnancy. Perinatal psychopharmacology may be constrained not only by reproductive safety considerations, but also by pregnancy-related symptoms that directly affect adherence and feasibility of treatment (13, 15). In the present case, discontinuation was driven primarily by persistent emesis and poor oral tolerability rather than by psychiatric recovery or the emergence of new safety concerns.

The postpartum course further illustrates that the risk-benefit balance may change across the perinatal period. During pregnancy, re-establishing milnacipran was considered justified because relapse had followed switching, illness burden was high, and maternal stabilization was clinically urgent. In contrast, postpartum re-initiation was not considered warranted, as the patient remained psychiatrically stable, adhered to regular follow-up, and had experienced unpleasant adverse effects. This single case cannot establish safety, but it illustrates the clinical challenge of balancing relapse risk, prior treatment response, and treatment feasibility in pregnancy when evidence for a specific agent is sparse. This underscores the need for more systematic data on milnacipran exposure and other understudied antidepressants during pregnancy and lactation to better inform individualized risk-benefit decisions in high-risk patients.

## Patient perspective

The patient reported that milnacipran was more effective than any previous medication, particularly in reducing suicidal symptoms and dissociative experiences. At the same time, she described feeling “being wrapped in cotton wool” and experiencing “brain fog” while taking it. Despite these adverse effects, she considered milnacipran highly beneficial during this phase of acute psychiatric crisis and felt that it helped stabilize her during pregnancy.

She further stated that, after delivery, she was “really positively surprised that despite no medication I didn’t develop postpartum depression, since my doctor warned me about the possible risks of relapse.” She reported that this stability continued during breastfeeding. She was especially relieved that her child remained well and has shown no developmental problems to date. Overall, she stated that she would consider taking milnacipran again as a “crisis medication,” but that the sedation and cognitive dulling she experienced would make it unsuitable for her as a long-term treatment option.

## Data Availability

The original contributions presented in the study are included in the article/**Supplementary Material**. Further inquiries can be directed to the corresponding author.
