# Supplementary material for: Milnacipran continuation during pregnancy in a patient with severe post-traumatic stress disorder and psychotic depression: a case report with 3-year pediatric follow-up
Source: Front Psychiatry. 2026 Apr 27;17:1830520. doi: 10.3389/fpsyt.2026.1830520 (PMC13158198; doi:10.3389/fpsyt.2026.1830520)
Supplement: Supplementary file 1 [file DataSheet1.pdf]

CARE case report guidelines

CARE Checklist of information to include when writing a case report

(cc) BY-NC-ND

Topic	Item	Checklist item description	Reported on Line
Title	1	The diagnosis or intervention of primary focus followed by the words "case report"	yes
Key Words	2	2 to 5 key words that identify diagnoses or interventions in this case report, including "case report"	yes
Abstract (no references)	3a	Introduction: What is unique about this case and what does it add to the scientific literature?	yes
	3b	Main symptoms and/or important clinical findings	yes
	3c	The main diagnoses, therapeutic interventions, and outcomes	yes
	3d	Conclusion—What is the main "take-away" lesson(s) from this case?	yes
Introduction	4	One or two paragraphs summarizing why this case is unique (**may include references**)	yes
Patient Information	5a	De-identified patient specific information.	yes
	5b	Primary concerns and symptoms of the patient.	yes
	5c	Medical, family, and psycho-social history including relevant genetic information	psychosocial history
	5d	Relevant past interventions with outcomes	yes
Clinical Findings	6	Describe significant physical examination (PE) and important clinical findings.	yes
Timeline	7	Historical and current information from this episode of care organized as a timeline	yes
Diagnostic Assessment	8a	Diagnostic testing (such as PE, laboratory testing, imaging, surveys).	yes
	8b	Diagnostic challenges (such as access to testing, financial, or cultural)	none reported
	8c	Diagnosis (including other diagnoses considered)	yes
	8d	Prognosis (such as staging in oncology) where applicable	non applicable
Therapeutic Intervention	9a	Types of therapeutic intervention (such as pharmacologic, surgical, preventive, self-care)	yes
	9b	Administration of therapeutic intervention (such as dosage, strength, duration)	yes
	9c	Changes in therapeutic intervention (with rationale)	yes
Follow-up and Outcomes	10a	Clinician and patient-assessed outcomes (if available)	not available
	10b	Important follow-up diagnostic and other test results	yes
	10c	Intervention adherence and tolerability (How was this assessed?)	yes
	10d	Adverse and unanticipated events	yes
Discussion	11a	A scientific discussion of the strengths AND limitations associated with this case report	yes
	11b	Discussion of the relevant medical literature **with references**.	yes
	11c	The scientific rationale for any conclusions (including assessment of possible causes)	yes
	11d	The primary "take-away" lessons of this case report (without references) in a one paragraph conclusion	yes
Patient Perspective	12	The patient should share their perspective in one to two paragraphs on the treatment(s) they received	yes
Informed Consent	13	Did the patient give informed consent? Please provide if requested	Yes ☑ No ☐
